# Cervical cancer screening programmes and policies in 18 European countries

**DOI:** 10.1038/sj.bjc.6602069

**Published:** 2004-07-27

**Authors:** A Anttila, G Ronco, G Clifford, F Bray, M Hakama, M Arbyn, E Weiderpass

**Affiliations:** 1 The Finnish Cancer Registry, Helsinki, Finland; 2 Unit of Cancer Epidemiology, CPO Piemonte, Torino, Italy; 3 The International Agency for Research on Cancer, Lyon France; 4 Scientific Institute of Public Health, Brussels, Belgium; 5 Cancer Registry of Norway, Oslo, Norway; 6 Department of Medical Epidemiology and Biostatistics, Karolinska Institutet, Stockholm, Sweden

**Keywords:** epidemiology, cervix uteri, screening, evaluation, monitoring

## Abstract

A questionnaire survey was conducted by the Epidemiology Working Group of the European Cervical Cancer Screening Network, and the International Agency for Research on Cancer, IARC, between August and December 2003 in 35 centres in 20 European countries with reliable cervical cancer incidence and/or mortality data in databanks held at IARC and WHO. The questionnaire was completed by 28 centres from 20 countries. The final tables included information on 25 centres from 18 countries. Six countries had started screening in the 1960s, whereas 10 countries or regions had started at least a pilot programme by 2003. There were six invitational and nine partially invitational programmes, the rest employing opportunistic screening only. Recommended lifetime number of smears varied from seven to more than 50. Coverage of smear test within the recommended screening interval (usually 3 or 5 years) was above 80% in three countries. Screening registration took place in 13 programmes. Eight programmes reported the rates of screen-detected cervical cancers and precursor lesions. There was wide variation in the CIN3 detection rates. International guidelines and quality assurance protocols are useful for monitoring and evaluating screening programmes systematically. Our survey indicated that the recommendations as currently given are met in only few European countries. Health authorities need to consider stronger measures and incentives than those laid out in the current set of recommendations.

## BACKGROUND

Organised screening programmes for cervical cancer using Pap smears have been shown to be effective in decreasing mortality and incidence from the disease (Fidler *et al*, 1968; Hakama and Räsänen-Virtanen, 1976; Hakama, 1982; Day, 1986; Läärä *et al*, 1987; Lynge, 2000). Opportunistic or nonorganised screening also decrease cervical cancer rates, although not to the extent of organised programmes (Magnus and Langmark, 1986; Lynge *et al*, 1989; Nieminen *et al*, 1999). One problem with nonorganised screening is that it may not adequately cover the population targeted, sometimes missing those at highest risk (Coleman *et al*, 1993a).

If clinical and diagnostic quality are not monitored and evaluated systematically, as in nonorganised screening, there are also concerns that adverse effects may become more common (Council of the EU, 2003). The goal of an organised programme, along with achieving reasonable effectiveness, is that potential adverse effects are minimised while screening-related improvements in the quality of life maximised. Overuse of services can be prevented and a complete evaluation can be implemented only within the framework of an organised programme.

The European Union (EU) currently recommends that cancer screening should only be offered on a population basis in organised screening programmes, with quality assurance at all levels (Council of the EU, 2003). There are also some more detailed recommendations describing the implementation, registration, monitoring, training, compliance, and introduction of novel tests of organised cancer screening programmes (Advisory Committee on Cancer Prevention, 2000; Sankila *et al*, 2000; Council of the EU, 2003;). Managerial guidelines have also been published by the WHO, and there are guidelines in several areas or individual countries describing how to organise a programme.

In Europe, there are wide variations in the organisation of cervical cancer screening activities (Linos *et al*, ed., 2000). The present study aims to describe the main policy and organisational elements in cervical cancer screening programmes in many European countries, and compare them with the EU and other recommendations.

## MATERIALS AND METHODS

The study is based on a questionnaire survey, collected in late 2003 from 20 European countries within the framework of a collaborative research project of the European Cervical Cancer Screening Network (ECCSN), funded by the Europe Against Cancer programme, and the International Agency for Research on Cancer (IARC), Lyon. The questionnaire survey was conducted in August–December 2003. The present report summarises the current and historical screening situation in Europe. However, as these data were also collected within the framework of interpreting long-term cervical cancer trends at a population level, only European countries or regions for which cervical cancer mortality and/or incidence data met eligibility criteria for the assessment of cervical cancer trends were included.

More specifically, countries or regions were selected according to the following criteria:

1. Countries with mortality data available for more than 10 years (not necessarily consecutively) in the WHO mortality database up to 2000, and where ‘Not Otherwise Specified’ uterine cancer deaths accounted for less than 25% of all uterine cancer deaths (these NOS uterine cancer deaths can be redistributed based on the age-specific proportion of registered cervix and corpus cancer deaths (Arbyn and Geys 2002; Bray *et al*, 2002)).
2. Countries without mortality data meeting the above quality criteria, but for which cervical cancer incidence data of sufficient quality was available, either nationwide, or within a specific region. Criteria for data quality consisted of cancer registry-based incidence data published in at least three consecutive volumes of ‘Cancer incidence in five continents’ from IARC (Parkin *et al*, 1992, 1997, 2002).

Countries meeting criteria for mortality data were: the Czech Republic, Denmark, Estonia, Finland, Hungary, Iceland, Lithuania, Luxembourg, the Netherlands, Norway, Switzerland, and the UK. Countries and regions meeting criteria for incidence data were: France (regional data only, from eight regions: Bas-Rhin, Calvados, Doubs, Herault, Isére, Limousin, Somme, Tarn); Germany (Saarland only); Italy (from four regions: Florence, Parma, Ragusa, Varese, and two cities: Genova, Torino); Poland (Cracow only); Slovakia, Slovenia, Spain (from four regions: Catalonia, Granada, Murcia, Navarra), and Sweden. For countries with only regional incidence data, region-specific information on cervical cancer screening was requested.

Emphasis was placed on collecting both current and historical information on the following:

- *Screening policy:* Year of programme initiation; target age range of screening; screening interval for women with normal results; financial cost of the smear to the women;
- *Organisational issues:* Whether all women in the target population are invited, or only those without a recent smear; the manner in which women are invited (personally or otherwise); the data source from which invitations are drawn; whether invitations and visits are centrally registered on an individual basis; if there had been, historically, important changes in the screening organisation;
- *Process and performance measures:* Invitational and geographical coverage of the programme or policy; screening attendance; proportion of women tested at least once within the recommended interval; availability of data on detection rates of histologically confirmed cancerous or precancerous findings, by severity of lesions.

We also enquired as to how estimates on screening parameters were collected and calculated in order to assess the reliability of the reported information, and we also searched for all published information on the programmes.

Different organisational definitions affect the applicability of the concept, while invitational coverage can apply only among invitational programmes. Another measure of coverage, the proportion of the target population subject to formal programme or policy (van Ballegooijen *et al*, 2000), was included in the tables. In addition, proportion of women tested at least once within the recommended interval was used. The latter attendance rate is a combined measure of invitational coverage and related attendance, plus noninvitational smear-taking activity.

In addition to smears taken within programmes, spontaneous smears taken outside the programme were reported by several centres. Lack of information prevented their inclusion in detail in all performance measures. For those programmes, which registered smears, proportions of women tested at least once during the recommended interval could be calculated from the register-based source. For those programmes that registered only the invitational programme, corresponding estimates were based usually on questionnaire surveys where the reliability of the information may be limited due to reporting and selection biases. Owing to the lack of information, calculations could not be carried out for some other relevant time windows, such as smears during the last 10 year period.

The 2003 survey was sent to 35 centres in 20 countries and was completed by 28 centres from 20 countries. In the returned questionnaires from Estonia, Cracow (Poland), and Somme (France), it was reported that no ‘organised programme or otherwise defined screening policy for cervical cancer’ existed and there were no responses to further questions on screening activities. Therefore, these countries/regions were not included in the detailed tables. All the questionnaire information was managed in a database at IARC.

## RESULTS

Details of screening policy are included in Table 1

**Table 1:** Screening policy in the 18 European countries

**Country/region**	**Onset of screening programme or policy**	**Age range targeted (since year)**	**Recommended screening interval**	**Charge of smear for the women**	**Recommended number of lifetime smears**
Czech Republic	1966	Not specified (1966)	1 year	Free	Not specified
Denmark	1967	23–59 (1986)	3 years (some counties 5 years in >45 or 50)	Free	13
Finland	1963	30–60 (1993)	5 years	Free	7
Hungary	2003, pilot 1997	25–65 (1997)	3 years, after one negative smear	Free	15
Iceland	1964	20–69 (1988)	2 years	Partial contribution (31 USD)	25
Lithuania	2001	30–60 (2001)	5 years	Free or partial contribution (5 euro)	7
Luxembourg	1962	15+ (1990)	1 year	Free	∼70
Netherlands	1980	30–60 (1996)	5 years	Free	7
Norway	1995, pilot 1992 (programme in one county in 1959–1977)	25–69 (1992)	3 years	Partial contribution	15
Slovakia	— (intention)	25–64 (—)	3 years	Free	14
Slovenia	2003 (1955 opportunistic)	20–64 (2002)	3 years	Free	15
Sweden	1967–1973 in different counties, Gothenburg 1977	23–60 (1999)	3 years in ages 23–50 years; 5 years in ages 51–60 years	Complete contribution in most counties; free or partial in some counties	12
Switzerland	No data	18–69 (—)	3 years	Partial contribution	18
UK	1988	20–64 (1988, under review)	3–5 years (currently 3 years in ages 25–49 years and 5 years in ages 50–64 years)	Free	10–17 (12)
France					
Bas-Rhin	1994	25–65 (1990)	3 years	Partial contribution	14
Doubs	1993	20–65 (1993)	3 years (after two normal exams with 1 year interval)	Reimbursement	17
Isére	1990	50–69 (1990)	3 years	Free	7
Germany					
Saarland	1971	20–85+ (1982)	1 year	Free	∼65
Italy					
Florence	1982	25–64 (1995)	3 years	Free	14
Genova	1992	25–64 (1992)	3 years	Free	14
Parma	1998	25–64 (1998)	3 years	Free	14
Ragusa	No data	25–64 (1996)	3 years	Free	14
Torino	1992	25–64 (1992)	3 years	Free	14
Varese	No data	25–64 (1996)	3 years	Free	14
Spain					
Catalonia	No data	20–64 (1993)	3–5 years: initially two smears 1 year apart. Then, 3 years in ages 20–34 years and 5 years in ages 35–64 years	Free or partial contribution	12

, irrespective of whether organised, spontaneous, or nonspecific screening programmes were in place. Six countries (the Czech Republic, Denmark, Finland, Iceland, Luxembourg, Sweden) reported having started screening in the 1960s, whereas 10 other countries or regions (Hungary, Lithuania, Norway, Slovenia, Bas-Rhin, Doubs, Isere, Genova, Parma, Torino) had started at least a pilot by 2003. There were large differences in target age range and interval. Recommended lifetime number of smears varied from seven (Finland, Lithuania, Netherlands, Isere) to more than 50 (Luxembourg, Germany).

The cost covered by the screened women varied widely. In most of the regions (17 out of 25; 68%), screening was free of charge for the women but in several programmes payment practices varied even within the programme, depending on the area or mode of screening activity.

Six countries/regions had invitational programmes (Finland, Hungary, the Netherlands, Isére, Parma, Torino), whereas nine countries/regions (Denmark, Iceland, Norway, Slovenia, Swdeden, United Kingdom, Bas-Rhin, Doubs, Florence) invited only those women who had not had a smear (usually opportunistic) recently or within the recommended interval (Table 2

**Table 2:** Organisation characteristics in screening for cervical cancer in the 18 European countries

**Country/region**	**Are women in the target population invited personally to participate?**	**How women are invited/smears offered**	**Source of personal invitations**	**Is screening registered on an individual basis?**	**Information available on screen-detected histological findings?**	**Remarks**
Czech Republic	No	Opportunity	—	No	No	
Denmark	Yes, only women without a recent smear	Letter or opportunity	Population, pathology and health insurance registries	No national registration. Varies between counties, most have all smears and histology in the county pathology register	No	Organised screening was introduced gradually county-wise. Information on screening and histological findings can be collected as a research activity
Finland	Yes	Letter	Population registry	Yes, centrally, invitational programme	Yes	Organised screening introduced gradually during 1963–1970; invitational coverage almost complete since 1971
Hungary	Yes	Letter	Social security register and health insurance funds	Yes, centrally, invitational programme	No	
Iceland	Yes, only women without a recent smear	Letter or opportunity	Population registry	Yes, centrally, all	Yes	Computerised call-recall system from 1964. About 70% of smears in 2000 were from the invitational programme
Lithuania	No (yes in some regions)	Opportunity, announcements, mass media	Health service registers	Yes, centrally (in 10 out of 60 regions), invitational programme	Yes	
Luxembourg	No	Opportunity	—	Yes, most of them at the national health laboratory	Yes	Reorganisations in 1980 and 1990
Netherlands	Yes	Letter	Population registry	Yes, both centrally and regionally, all	Yes	
Norway	Yes, only women without a recent smear	Letter or opportunity	Population registry	Yes, centrally, all	Yes	
Slovakia	No (yes in some districts)	Letter or opportunity	Health service registers	No	No	
Slovenia	Yes, only women without a recent smear	Letter or opportunity	Population and health service registers	Yes, centrally, all	Yes (under construction)	
Sweden	Yes, only women without a recent smear	Letter or opportunity	Population registry	Yes, regionally, all. A central register under construction	Yes (under construction)	
Switzerland	No	Opportunity	—	—	No	
UK	Yes, only women without a recent smear	Letter or opportunity	Health services register	Yes, centrally and regionally, all	Yes	Computerised call-recall in 1988. Target incentive payments to GPs since 1990. National coordination and quality assurance introduced in 1995
France						
Bas-Rhin	Yes, only women without a recent smear	Letter or opportunity	Health service register	Yes, regionally, all	Yes	
Doubs	Yes, only women without a recent smear	Letter and/or campaign	Social security register	Yes, regionally, all	No data	
Isére	Yes	Letter	Social security register	Yes, regionally, invitational programme	No data	Pap smear screening in connection with breast cancer screening
Germany						
Saarland	No	Smears offered through health insurance scheme	—	No	No	
Italy						
Florence	Yes, only women without a recent smear	Letter or opportunity	Population registry	Yes, regionally, all	Yes	
Genova	No	Opportunity	—	No	No	
Parma	Yes	Letter	Population and health service register	Yes, regionally, invitational programme	Yes	
Ragusa	No	Opportunity	—	No	No	
Torino	Yes	Letter	Population registry	Yes, regionally, invitational programme	Yes	
Varese	No	Opportunity	—	No	No	Screening campaigns in the past within part of the area
Spain						
Catalonia	No	Opportunity	—	No	Yes	

). The other regions did not invite women, but screening was offered mainly at the occasion of a visit to a general practitioner or gynaecologist.

All of the six fully invitational programmes also had a centralised national or regional screening registration database arranged on an individual basis. Five of these registers included only the invitational programme and one (the Netherlands) included any smears. From among the programmes with partial invitations, two programmes (Denmark, Sweden) did not have a centralised national registration unit. The rest of the national programmes with a partial invitational activity reported a central national registration system, and these registers aimed to include all smears, whether taken after invitation or otherwise. Of the 10 countries/regions with opportunistic screening policies, eight were without screening registration and two had partial registration.

Smear test coverage above 80% of the target population during the recommended screening interval was reported in three national programmes (Finland, Sweden, UK); and the smear coverage was 75–80% in three countries (Denmark, Iceland, the Netherlands).

A total of 11 programmes reported collection of information on histologically confirmed lesions (Table 2). Six of these programmes reported detection rates by grade of the histological finding (Finland, Iceland, Norway, Bas-Rhin, Florence, Torino) and one country (the Netherlands) did not separate invasive cancers from the CIN3 (Table 3

**Table 3:** Process and performance values in screening for cervical cancer in the 18 European countries

			**Detection rate (%)**		
**Country/region**	**Population subject to formal programme (in ages 25–64 years unless specified)**	**Women screened within recommended interval (%), any smears included (in ages 25–64 years in 2000 unless specified)**	**Invasive (ICC)**	**CIN3**	**CIN1-2**
Czech republic	No data	No data	No data	No data	No data
Denmark	90% (23–59)	75% (23–59, 1997)	No data	No data	No data
Finland	100% (30–60)	93% (35–64, 1997)	0.01	0.13	0.21
Hungary	No data	30%	No data	No data	No data
Iceland	100%	76% within a 3-year interval (25–69)	0.015	0.47	0.27
Lithuania	No data	No data	No data	No data	No data
Luxembourg	No data	39% (1 year, 1999)^a^	No data	No data	No data
Netherlands	100% (30–60)^b^	77% (30–60, 1997)^b^	No data	0.35 (incl. CIN3+)	0.13
Norway	100%	70%	0.04^c^	0.50^c^	0.79^c^
Slovakia	No data	No data	No data	No data	No data
Slovenia	31% in 2000	70%	No data	No data	No data
Sweden	100%^b^	83 (23–59; 1997)^b^	No data	No data	No data
Switzerland	No data	No data^d^	No data	No data	No data
UK	100%	83%	No data	No data	No data
France					
Bas-Rhin	No data	69%^e^	0.05	0.35	0.73
Doubs	88%	>50%	No data	No data	No data
Isére	No data	69% (50–69, 1996)	No data	No data	No data
Germany					
Saarland	90%^b^	50% per year (20+; 1996); 80% within 3 years^b^	No data	No data	No data
Italy					
Florence	100%	73%	0.008	0.13	0.12
Genova	No data	53%^f^	No data	No data	No data
Parma	100%	66%	No data	No data	No data
Ragusa	No data	53%^f^	No data	No data	No data
Torino	100%	>74%	0.011	0.09	0.14
Varese	No data	53%^6^	No data	No data	No data
Spain					
Catalonia	No data	No data	0.04	0.06	0.8

a From Scheiden *et al* (2000).

b From Van Ballegooijen *et al* (2000); whole Germany.

c In 1998, from The Cancer Registry of Norway (2001).

d 80% ever-screened (18–75; 1997).

e From Schaffer *et al* (2000).

f No regional data. Italian national estimate 1999/2000.

). Histological information was also reported from Catalonia, a region with opportunistic activity only. No published routine monitoring information was available for other programmes. There was a wide variability in the rates between the seven programmes from 0.008 to 0.04% for invasive cancer, from 0.06 to 0.50% for CIN3, and from 0.12 to 0.8% for CIN1-2. CIN3 : invasive cancer detection ratios ranged from 1.5 to 12.

## DISCUSSION

The European Union has currently recommended that cancer screening should only be offered on a population basis in organised screening programmes, with quality assurance at all levels (Council of the EU, 2003). There are also more detailed recommendations that are valuable in relation to organisational aspects (Advisory Committee on Cancer Prevention, 2000; Council of the EU, 2003). The present questionnaire-based survey indicates that these recommendations are met in only a few European countries.

The most serious inadequacies relate, according to our survey, to: (1) low or inadequate coverage of the screening test within the populations targeted; (2) shortcomings in routine registration, evaluation, and monitoring; and (3) excessive numbers of lifetime smears recommended. There also exist relatively short screening intervals that are not justified as present knowledge of the natural history of cervical cancer, particularly on the duration of precancerous stage. Such aspects as payment strategies, possibly affecting attendance, varied greatly, indicating a potential for inequality.

Incidence and mortality rates from cervical cancer can be decreased by at least 80% by means of screening. This has been shown from follow-up studies of women screened negative (Day, 1986), cohort follow-up studies among screened women (Fidler *et al*, 1968; Hakama and Räsänen-Virtanen, 1976; Lynge, 2000), and population-based follow-up studies (Hakama 1982; Day, 1986; Läärä *et al*, 1987). Declines of this order have been observed in Canada (British Columbia) (Anderson *et al*, 1988) and in Finland and Iceland (Läärä *et al*, 1987; Sigurdsson, 1999; Anttila and Läärä, 2000). In the other Nordic countries, decreases of between 40 and 60% have been reported (Sigurdsson, 1999), while the reduction tends to be of a somewhat lower order of magnitude in other regions and countries (Coleman *et al*, 1993b; Beral *et al*, 1994). Information is variable and often very limited, however, concerning the screening activities or incidence or death rates before the assumed time that screening started. These data as well as the current estimates of cervical cancer in Europe (Bray *et al*, 2002) suggest that meaningful additional decreases in the cervical cancer rates are still possible. It is important therefore to utilise the available data continually to monitor cervical incidence and mortality rates in these populations.

The current data were obtained from areas covered by long-standing cancer registries. Therefore, they may not be representative of the entire European situation. Frequently there is a link between pilot programmes for cervical cancer screening and cancer registration, given the utility of the latter using planning and screening evaluation. For example in France, all three existing regional programmes were in areas with a cancer registry considered to satisfy minimal quality assurance prerequisites. The study may thus overestimate the presence of organised programmes.

The reported CIN3 detection rates varied eight-fold. This can be expected to result in *t* differences in related treatment rates. These differences are apparently not explained by differential screening intervals or age ranges. Variation in the background risk could provide a partial explanation, but the variations in cancer to CIN3 detection ratios suggest that differential diagnostic and registration criteria may play a major role.

The disadvantages of cancer screening include: anxiety among those screened positive or treated for a lesion, complications, potential of unnecessary medical interventions (e.g. among false-positives), overtreatment of questionable or nonprogressive abnormalities, costs incurred, longer morbidity for cases whose prognosis is unaltered, and also false reassurance that can result in delayed presentation or investigation of symptoms for persons with false-negative test results (Hakama, 1991; Bennetts *et al*, 1995; Lauver *et al*, 1999; Peters *et al*, 1999; Rogstad, 2002; Idestrom *et al*, 2003). Quality-of-life and potential adverse aspects should be included in the evaluation of the screening programmes. These also represent important aspects to be considered for any potential new screening techniques to be implemented in programmes.

Several Eastern European countries, which had established cancer registries, were included in this questionnaire study, but most had not implemented an organised screening programme. It should be noted that mortality rates are uniformly increasing in several countries in this region (Beral *et al*, 1994; IARC, 2002). Whenever possible, cancer registries should be involved in the planning and monitoring of screening programmes. Availability of local resources needs to be carefully taken into account. Given limited screening resources, the programme should be started in a few age groups, with high coverage being prioritised.

In general, there are large variations in European cervical cancer screening policies and in the organisation of programmes. Limited details are available on process and performance measures, and their correlation with effectiveness is not known. In particular, registration, monitoring, and evaluation are deficient. The EU Council recommendation states that ‘subsequent monitoring and data collection on the extent to which the proposed measures are working effectively need to be arranged for the next 3-year period’. Decision-makers and health-care service providers need to consider stronger measures and incentives than the current recommendations in order to deliver successful cervical cancer control in Europe.

## Appendix

**Questionnaire survey in Cervical Cancer Screening in Europe: List of contributors**

The Czech Republic: Vlasta Mazankova, Czech Cancer Registry, Prague. Denmark: Elsebeth Lynge, University of Copenhagen, Copenhagen. Estonia: Mati Rahu, Estonian Cancer Registry, Tallinn. Finland: Ahti Anttila, Finnish Cancer Registry, Helsinki. France: (Bas-Rhin) Muriel Fender, Association Eve, Strassbourg; (Doubs) Muriel Fender, and Christine Bouiller, Association pour Prévention du Cancer du Col dans le Doubs; (Isère) Muriel Fender, and Annie Garnier, ODLC, Meylan; (Somme) Muriel Fender, and Nicole Raverdy, Somme Cancer Registry, Amiens. Germany: Hartwig Zeigler, Saarland Cancer Registry, Saarbrucken. Hungary: Lajos Döbrössy and Miklos Bodo, St John Hospital, Budapest. Iceland: Kristjan Sigurdsson, Icelandic Cancer Society, Reykjavik. Italy: (Genova, Ragusa, Torino, Varese) Guglielmo Ronco, CPO Piemonte, Torino; (Parma) Guglielmo Ronco, and Carlo Naldoni, Region Emilia-Romagna; (Florence) Marco Zappa, (CSPO Florence), and Guglielmo Ronco. Lithuania: Juozas Kurtinaitis, Lithuanian Cancer Registry, Vilnius. Luxembourg: Astrid Scharpantgen, Ministry of Health, Luxembourg. The Netherlands: Marjolein van Ballegooijen, Erasmus University, Rotterdam. Norway: Steinar Thoresen, Cancer Registry of Norway, Oslo. Poland: (Cracow) Jadwiga Rachtan, Cracow Cancer Registry, Cracow. Slovakia: Ivan Plesko, National Cancer Registry of Slovak Republic, Bratislava. Slovenia: Maja Zakelj, Cancer Registry of Slovenia, Ljubljana. Spain: (Catalonia) Xavier Castellsagué, Institut Catalá d'Oncologia, Barcelona. Sweden: Pär Sparen, Karolinska Institute, Stockholm. Switzerland: Felix Gurtner Bundesamt fur Sozialversicherungen, Bern. The UK: Julietta Patnick, National Health Service Cervical Screening Programme, Sheffield.
